# The Lower Urinary Tract Science Group Abstracts—ICI‐RS 2024

**DOI:** 10.1002/nau.25643

**Published:** 2024-12-29

**Authors:** Christopher H. Fry, Andrew Gammie, Bahareh Vahabi, Faezeh A. Hassani, Karen D. McCloskey, Mathijs M. de Rijk, Pradeep Tyagi, Hikaru Hashitani, Michael Winder, Patrik Aronsson, Basu Chakrabarty

**Affiliations:** ^1^ School of Physiology, Pharmacology & Neuroscience University of Bristol Bristol UK; ^2^ Bristol Urological Institute, Southmead Hospital North Bristol NHS Trust Bristol UK; ^3^ School of Applied Sciences University of the West of England Bristol UK; ^4^ School of Electrical, Electronic and Mechanical Engineering University of Bristol Bristol UK; ^5^ Patrick G Johnston Centre for Cancer Research Queen's University Belfast Belfast UK; ^6^ Department of Urology Institute for Mental Health and Neuroscience Maastricht University Maastricht The Netherlands; ^7^ Department of Medicine University of Pittsburgh Pittsburgh Pennsylvania USA; ^8^ Department of Cell Physiology Nagoya City University Nagoya Japan; ^9^ Department of Pharmacology University of Gothenburg Gothenburg Sweden

**Abstract**

**Aims:** The aims of this preliminary session of the International Consultation on Incontinence‐Research Society (ICI‐RS) were to provide a forum for an international group of experimental scientists to explain their on‐going work to fellow laboratory scientists, to obtain feedback about future directions and to discuss potential future collaborations.

**Methods:** Individual abstracts of the associated talks were prepared by the attendees.

**Results:** Eleven presentations and associated abstracts were presented.

**Conclusions:** The presentations and attendant abstracts reflect a wide variety of fundamental research currently underway and are designed to have translational outputs with respect to the management and treatment of lower urinary tract pathologies.

**KEYWORDS:**

abstracts, lower urinary tract, translational research

**Investigating the relative phase of abdominal pressure and urine flow rate in male patients**

**Andrew Gammie** ^**1**^, **Elisabeth Kirton** ^**2**^

^1^Bristol Urological Institute, Southmead Hospital, Bristol BS10 5NB, UK. ^2^Department of Urology, Hull University Teaching Hospital, UK.

**Introduction:** Straining to void is unproductive for men with bladder outflow obstruction (BOO)^1^ and it has a higher likelihood of occurring in patients with detrusor underactivity (DU).^2^ We investigated if analysis of abdominal strain and urine flow could be used as a diagnostic test.

**Methods:** We gathered data from invasive urodynamics carried out in men who showed straining of more than 10 cmH_2_O on the abdominal pressure (p_abd_) line during their pressure flow study. We assessed maximum changes in p_abd_ and flow rate (Q), and the fastest rise in urine flow (dQ/dt), measuring the corresponding rate of change in p_abd_ (dp_abd_/dt) at that time.

**Results:** The urodynamic voiding data for nine Normal voiders, 10 BOO and nine DU men were found to include straining of more than 10 cmH_2_O. The changes in Q, and the ratio of p_abd_ to Q changes, between BOO and Normal groups were statistically significantly different, but there was no difference between the DU and BOO groups. However, when considering the average gradient of p_abd_ during maximum Q change, the BOO group was statistically significantly different from both Normal and DU groups, suggesting its use as a diagnostic indicator.

**Conclusions:** We present analysis of trace gradients and phase during pressure‐flow studies. We have shown in this pilot study that when taken during the maximum flow rate rise, an abdominal pressure gradient of less than −3 cmH_2_O/s will indicate BOO with a sensitivity of 70% and a specificity of 94%.

**References:**

1. Reynard JM, Peters TJ, Lamond E, Abrams P. The significance of abdominal straining in men with lower urinary tract symptoms. *Br J Urol* 1995; 75: 148–153.

2. Gammie A, Kaper M, Dorrepaal C, Kos T, Abrams P. Signs and symptoms of detrusor underactivity: an analysis of clinical presentation and urodynamic tests from a large group of patients undergoing pressure flow studies. *Eur Urol* 2016; 69: 361–369.

**Ethical Approval**: Not applicable

**Funding:** None

**The effect of SARS‐CoV‐2 on lower urinary tract function**

**Bahareh Vahabi, Pravina Kuppanda, R. Cousins, V. Garner, C. Jones, Christopher H Fry, Paul White, Nikki Cotterill.**

School of Applied Sciences, University of the West of England, Bristol, UK.

**Introduction:** Severe Acute Respiratory Syndrome Coronavirus 2 (SARS‐CoV‐2), responsible for the COVID‐19 pandemic, continues to infect thousands globally. While respiratory distress remains the primary reason for hospital admission, SARS‐CoV‐2 also manifests in other tissues, including the liver, kidneys, gut, and heart. Emerging studies indicate that SARS‐CoV‐2 can lead to significant lower urinary tract symptoms (LUTS) such as urinary urgency, frequency, and nocturia. This study aimed to evaluate the impact of SARS‐CoV‐2 infection on the development, severity, and time‐course of LUTS in COVID‐19 patients.

**Methods:** Thirty‐two COVID‐19 positive (15 females, 17 males; 60–89 years) and 22 COVID‐19 negative (13 females, nine males; 60–89 years) subjects was recruited with consent from North Bristol NHS Trust, UK, between March 2022 and February 2023. LUTS and bothersomeness was assessed at baseline (1‐month posthospital discharge for COVID‐19 positive patients or at recruitment for COVID‐19 negative patients) and after 6 months, using validated questionnaires (ICIQ‐F/MLUTS). The prevalences of urgency, frequency and nocturia symptoms were compared between groups and data analyzed using chi^2^‐tests of association.

**Results:** Preliminary data indicated a significantly (*p* < 0.05) higher incidence of severe nocturia (two or more voids per night) in COVID‐19 positive patients (84.4%) compared to COVID‐19 negative patients (59.1%) of the same age range. This difference was evident at baseline but not at 6 months postinfection. There were no significant differences between COVID‐19 positive and negative patients in terms of urinary frequency or urgency symptoms at both baseline and 6 months postinfection.

**Conclusions:** These preliminary results suggest that COVID‐19 patients exhibit a higher incidence of nocturia up to 1‐month postinfection, compared to age‐matched controls, that did not persist after 6 months. Daytime urinary urgency or frequency were unaffected at either time‐point.

**Ethical Approval:** IRAS 299178

**Funding:** North Bristol NHS Trust

**Advances in assistive devices for dysfunctional bladders**

**Faezeh Arab Hassani**

School of Electrical, Electronic and Mechanical Engineering, University of Bristol, 75 Woodland Rd, Bristol BS8 1UB, UK.

**Introduction:** The development of an assistive solution for a neurogenic underactive bladder (UAB) with lost sensation and weak muscle control is challenging due to the bladder's delicate properties and extreme changes in volume during urine storage and voiding. Several wearable and minimally invasive technologies have been developed for bladder volume detection.^1,2^ However, assisting the bladder muscle in voiding is more challenging and requires implantable solutions. Bladder muscle electrical and optogenetic stimulation, bladder actuators, and artificial bladders are a few examples of explored voiding technologies.^1^ Since replacing the bladder with an artificial organ requires long‐term efficacy and compatibility testing, and direct stimulation of the bladder muscle lacks the required voiding efficiency, assistive actuators for physical compression of the bladder muscle are the next potential solution for emptying the bladder.

**Methods:** Several bladder‐inspired sensing‐actuation devices have been developed for UAB.^3^ The assistive devices include a sensor suitable for real‐time bladder volume monitoring and an actuator that can empty the bladder upon activation following a full bladder status.

**Result:** The most recent assistive device consists of a soft sensor suitable for continuous interaction with the delicate bladder tissue, whereas the actuator shows compliance with the bladder volume changes with the highest voiding efficiency of 100%.^3^

**Conclusion:** The sensing‐actuation systems represent an efficient voiding solution that avoids overfilling by real‐time monitoring of the bladder volume and allows on‐time complete emptying of the bladder upon the user's request.

**References:**

1. Holmes‐Martin K, Zhu M, Xiao S, Hassani FA. Advances in assistive electronic device solutions for urology. *Micromachines* 2022; 13: 551.

2. Hassani FA. Bioreceptor‐inspired soft sensor arrays: recent progress towards advancing digital healthcare. *Soft Science* 2023, 3: 4, 31.

3. Hassani FA, Jin H, Yokota T, Someya T, Thakor NV. Soft sensors for a sensing‐actuation system with high bladder voiding efficiency. *Science Advances* 2020; 30: 18, eaba0412.

**Ethical Approval:** Not applicable

**Funding:** None

**Radiation and bladder function: In vivo study using a small animal radiation research platform**

**Niamh McKerr, Conor Breen, Kirtiman Srivastava, Daniel Crummey, Karl Butterworth, Michaela Pettigrew, Joe O'Sullivan, Kevin M Prise, Karen D McCloskey**

Patrick G Johnston Centre for Cancer Research, School of Medicine, Dentistry and Biomedical Sciences, Queen's University Belfast, Northern Ireland, UK.

**Introduction:** Radiation therapy for pelvic malignancies unavoidably impacts the urinary bladder causing lower urinary tract symptoms in 40%–50% of patients.^1^ Recent advances in preclinical irradiation modalities include the Small Animal Radiation Research Platform (SARPP) which delivers CT‐guided, defined radiation doses to rodents, mimicking the clinical setting.^2,3^ The aim of the present study was to investigate SARRP‐irradiation on mouse voiding patterns and bladder contractility.

**Methods:** Adult C57Bl/6 female mice received irradiation (IRR, 20 Gy) to the bladder under CT‐image‐guided SARRP protocols. Pre‐ and post‐IRR, mice were housed in cages for voided spot assay (VSA) of urination patterns. The number of void spots, void spot volume and void location were measured. Contractility of bladder strips was assessed by in vitro tension recordings^.4^

**Results:** Post‐IRR at 2‐week, 1‐month and 2‐month timepoints, around 50% of mice had increased void spot locations. This was less apparent at 3‐month, 4‐month and 6‐month timepoints. Two weeks post‐IRR, 50% of mice (*N* = 13/26) had an increased number of void spots (*p* < 0.01), decreased mean spot volume (*N* = 17/26, *p* < 0.05) and decreased maximum void spot volume (*N* = 18/26, *p* < 0.01). The 2‐week cohort exhibited an increased number of small volume (< 50 μL) and reduced number of large volume void spots (< 100 μL), versus pre‐IRR. Electrical field stimulation evoked neurogenic contractions that were significantly smaller (0.5–32 Hz) 2‐weeks post‐IRR, and gradually recovered to around 50% of pre‐IRR amplitude by 6‐month post‐IRR.

**Conclusions:** SARRP irradiation acutely increased the number of void spots and decreased void spot volume. This was consistent with impaired neurogenic contractions which might limit the ability to void.

**References:**

1. Browne C, Davis NF, Mac Craith E, Lennon GM, Mulvin DW, Quinlan DM, et al. A narrative review on the pathophysiology and management for radiation cystitis. *Adv Urol* 2015: 346812, 2015.

2. Groves AM, Paris N, Hernady E, Johnston CJ, Aljitawi O, Lee YF, Kerns SL, Marples B. Prevention of radiation‐induced bladder injury: a murine study using captopril. *Int J Radiat Oncol Biol Phys* 2023; 115: 972‐982.

3. Zwaans BM, Krueger S, Bartolone SN, Chancellor MB, Marples B Lamb LE. Modeling of chronic radiation‐induced cystitis in mice. *Adv Rad Oncol* 2016; 1: 333‐343.

4. McDonnell BM, Buchanan PJ, Prise KM, McCloskey KD. Acute radiation impacts contractility of guinea‐pig bladder strips affecting mucosal‐detrusor interactions. *PLoS One* 2018; 13: e0193923.

**Ethical Approval:** Animals (Scientific Procedures) Act 1986, UK; licence PPL #2826.

**Funding:** Medical Research Council (UK) MR/M012425/1.

**Translational investigation of periaqueductal gray sub‐region activity during urine storage**

**Mathijs M de Rijk** ^**1,2**^, **Gommert A van Koeveringe** ^**1**^, **Anne MJ Verstegen** ^**2**^

^1^Department of Urology, Institute for Mental Health and Neuroscience, Faculty of Health, Medicine and Life Sciences, Maastricht University, The Netherlands. ^2^Department of Nephrology, Harvard Medical School/Beth Israel Deaconess Medical Center, Boston, MA, USA.

**Introduction:** The periaqueductal gray (PAG) is suggested to be involved in both urine storage and voiding, functioning as a relay station between the lower urinary tract (LUT) and spinal cord and higher cortical and subcortical areas.^1^ The PAG receives bladder afferent information from the spinal cord, and neuroimaging suggests that PAG activity is altered in conditions with LUT sensory processing such as overactive bladder. The PAG consists of functional subunits which contribute differently to storage and voiding of urine.^2^ Ventrolateral PAG (vlPAG) neurons have direct connections with the pontine micturition center (PMC) and their inhibitory signaling may prevent activation of the PMC before the initiation of micturition is desired. Here, we combine neuroimaging in humans and chemogenetic experiments in mice to investigate activity in different functional areas in the PAG during storage.

**Methods:** 7‐Tesla resting‐state neuroimaging during empty and full bladder states in healthy adult women was used to map the PAG. We evaluated changes in functional connectivity between subunits of the PAG and the PMC during bladder filling. In mice, we used chemogenetics to silence neuronal activity in vlPAG GABAergic neurons.

**Results:** Neuroimaging indicates that PAG subunits exhibit unique changes in functional connectivity with the PMC during bladder filling. Chemogenetic mouse experiments reveal that silencing of GABAergic signaling in vlPAG induces urine loss in mice, suggesting that these neurons are essential for optimal urine storage.

**Conclusions:** Integration of human neuroimaging experiments with chemogenetics in mice facilitates an improved understanding of the neuroanatomy of the PAG across species and how different areas of the PAG contribute to LUT control.

**References:**

1. de Groat WC, Griffiths D, Yoshimura N. Neural control of the lower urinary tract. *Compr Physiol* 2015; 5: 327‐396.

2. Zare A, Jahanshahi A, Rahnama'i MS, Schipper S, van Koeveringe GA. The role of the periaqueductal gray matter in lower urinary tract function. *Mol Neurobiol* 2019; 56: 920‐934.

**Ethical Approval:** Local medical ethical committee (METC) and IACUC

**Funding:** Faculty of Health, Maastricht University, NL and NIH‐NIDDK‐DK125708

**Diffusion across the “water‐tight” bladder lining depends on size and gradient.**

**Pradeep Tyagi** ^**1**^, **Irina Zabbarova** ^**2**^, **Youko Ikeda** ^**2**^, **Anthony J Kanai** ^**2**^

Departments of Urology^1^ and Medicine^2^, University of Pittsburgh, Pittsburgh, PA, USA.

**Introduction:** The belief of a “water‐tight” lining in mammalian bladder for urine storage, with a minimal metabolic expenditure, is congruent with an asymmetric unit membrane and uroplakins on the apical surface of large umbrella cells in the urothelium to restrict the transcellular diffusion of solute bound water; the absence of aquaporin channels also precludes the facilitated diffusion of solute‐free water from lumen into urothelium. However, this “water‐tight” dogma is challenged by osmolality oscillations of stored urine^1^ and intravesical absorption of tritiated water^2^.

**Methods and Results:** Here, we theorized that the apico‐lateral umbrella cell borders lined with tight junctions permit passive paracellular diffusion of molecules as spheres of Stokes‐Einstein radius (*r*). Therefore, the diffusion rate of free and bound water complies with first order kinetics. Indeed, the threefold higher systemic uptake of water bound to Na^+^ ions (*r* = 1.3 Angstrom) than water bound to threefold larger glucose (*r* ≈ 3.3 Angstrom) from bladder conforms to Stokes‐Einstein diffusion^1,3^. While a net movement of free water across mammalian urothelium from isotonic fluids is antithetical to osmosis^1,3^, a higher urine osmolality^4^ increasing the systemic uptake of solute bound water is consistent with a downhill concentration gradient driving Fick's diffusion in compliance with first order kinetics.

**Conclusions:** We deduced that urine reabsorption via passive paracellular diffusion precedes the facilitated diffusion of water and of dissolved solutes via aquaporins, Na^+^ channels and urea transporters, expressed on the intermediate cell layer of urothelium. These channels and transporters also respond to the endocrine signaling for homeostatic adjustment of bladder capacity and plasma osmolarity during sleep^1^ and awake states.

**References:**

1. Kanai A, Everaert K, Apostolidis A, Fry C, Tyagi P, Van Huele A, et al. A better understanding of basic science may help our management of LUTS/LUTD in older persons with nocturnal polyuria and nocturia: ICI‐RS 2024. *Neurourol Urodyn*. 2024. doi:10.1002/nau.25565.
2. Hilson AJ, Lewis CA, Harland SJ. The permeability of the human bladder to water assessed using tritiated water. *Contrib Nephrol*. 1990; 79: 41–44.
3. Tyagi P, Ganguly A, Foley L, Hitchens T, Zabbarova I, Ikeda Y et al. Why “ water‐tight” bladder is averse to osmosis but amenable to diffusion? *Continence*. 2024; 11: in press.
4. Spector DA, Deng J, Stewart KJ. Hydration status affects sodium, potassium, and chloride transport across rat urothelia. *Am J Physiol Renal Physiol*. 2013; 305: F1669–F1679.

**Ethical Approval:** Not applicable

**Funding:** NIH DK108397

**Age‐related changes in the external urethral sphincter—A female mouse model**

**Hikaru Hashitani, Retsu Mitsui**

Department of Cell Physiology, Graduate School of Medical Sciences, Nagoya City University, Japan.

**Introduction:** Age‐related deterioration of the external urethral sphincter (EUS) as a manifestation of sarcopenia^1^ is a major pathogenesis of stress urinary incontinence^2^. Since appropriate animal models are required to explore mechanisms underlying age‐related EUS dysfunction, morphological changes in aged female mouse EUS were investigated.

**Methods:** Fluorescence immunohistochemistry was used to visualize fast striated muscle fibers using an anti‐fast myosin skeletal heavy chain antibody, neuromuscular junctions using a combination of antibodies against synaptophysin (synaptic terminals) and α‐bungarotoxin (nicotinic acetylcholine receptors (AChRs)), fibroblasts using PDGFRα‐GFP mice, capillary (vascular) network using CD31, an endothelial marker, or pericytes using NG2‐DsRed mice.

**Results:** In EUS of young adult mice (3–7 months), postsynaptic AChRs were well‐occupied on synaptic terminals of α‐motor neuron axons, while some postsynaptic AChRs were incompletely occupied by synaptic terminals in EUS of aged mice (18–20 months), indicating partial denervation in aged EUS muscle fibers^3^. There was no notable change in the density or morphology of fast muscle fibers between the two groups. Capillary density was significantly reduced in aged EUS compared with young EUS (*n* = 5, *p* < 0.05) associated with a reduced tendency of capillary pericytes (*n* = 4, *p* = 0.072), indicating capillary rarefaction in aged EUS^3^. The density of fibroblasts in the EUS was significantly higher in aged mice than young mice (*n* = 5, *p* < 0.05), indicating fibrotic change in the aged EUS.

**Conclusions:** In addition to partial denervation of EUS muscle fibers, capillary rarefaction and associated fibrosis may also contribute to age‐related EUS dysfunction as a local pathogenesis.

**References:**

1. Carlile A, Davies I, Rigby A, Brocklehurst JC. Age changes in the human female urethra: a morphometric study. *J Urol* 1988: 139: 532–535.
2. Hunskaar S, Lose G, Sykes D, Voss S. The prevalence of urinary incontinence in women in four European countries. *BJU Int* 2004; 93: 324–330.
3. Larsson L, Degens H, Li M, Salviati L, Lee YI, Thompson W et al. Sarcopenia: Aging‐related loss of muscle mass and function. *Physiol Rev* 2019: 99: 427–511.

**Ethical Approval:** Experimental Animal Committee NCU, H30M‐04.

**Funding:** JSPS KAKENHI 23K08767

**Adreno‐muscarinic contractile synergy of human hyperplastic prostate tissue**

**Ben Blake‐James** ^**1**^, **Basu Chakrabarty** ^**2**^, **Christopher H Fry** ^**1,2**^

^1^Department of Surgery, University College, London. ^2^School of Physiology, Pharmacology & Neuroscience, University of Bristol, UK.

**Introduction:** The smooth musculature of the lower urinary tract, receives primary functional innervation from either one arm of the autonomic nervous system (e.g., urethra, sympathetic; detrusor, parasympathetic) or from both arms (e.g., trigone).^1^ With the trigone, tension generated by simultaneous exposure to an adrenergic and muscarinic receptor is greater than the sum of responses when introduced independently, that is, synergy.^2^ Contractile synergy is also recorded in normal human prostate tissue.^3^ However, the situation with equivalent tissue from men with benign prostatic hyperplasia (BPH) is unknown.

**Methods:** Tissue strips (≤ 1 mm diam) were dissected from prostate “chips” resected at transurethral resection of the prostate from men (69 ± 9 (SD) yr) with BPH, superfused at 36°C and isometric contractions recorded. Data are mean ± SEM; *n *= number of strips.

**Results:** Phenylephrine (alpha‐agonist) and carbachol (cholinergic‐agonist) generated contractions with similar potency (5.36 ± 0.09; 5.58 ± 0.07, *n* = 29;9) but different potencies (phenylephrine > carbachol). In the presence of phenylephrine the carbachol response was augmented to 203 ± 8.0% (*n* = 114) of control (=100%). Augmentation of the response was independent of the magnitude of outflow tract obstruction (BOO); acute urinary retention, or International Prostate Symptom Score (IPSS) score. Augmentation was completely abolished by 4‐DAMP (M3‐selective muscarinic antagonist, pIC_50_ = 7.82 ± 0.50, *n* = 8), but only partially and less potently by pirenzepine (M1/2 antagonist; pIC_50_ = 6.41 ± 0.62, *n* = 5) or gallamine (nonspecific cholinergic antagonist; pIC_50_ = 6.52 ± 0.58, *n* = 5).

**Conclusions:** Human hyperplastic prostate synergy displays adreno‐muscarinic contractile synergy; the muscarinic receptor is the M3 subtype. A low‐dose combination of an alpha‐blocker and M3‐antagonist may be indicated to reduce BPH symptoms associated with BOO.

**References:**

1. Roosen A, Wu C, Sui GP, Fry CH. Synergistic effects in neuromuscular activation and calcium‐sensitization in the bladder trigone. *BJU Int* 2008; 101: 610–614.

2. Roosen A, Fry CH, Sui G, Wu C. Adreno‐muscarinic synergy in the bladder trigone: calcium‐dependent and ‐independent mechanisms. *Cell Calcium* 2009; 45: 11–17.

3. Walther S, Strittmatter F, Hennenberg M, Gratzke C, Stief CG, Roosen A. Adreno‐muscarinic synergy in the male human urinary outflow tract. *Neurourol Urodyn* 2018; 37: 2128–2134.

**Ethical Approval:** LREC London (R&WF), 2004/209

**Funding:** The Prostate Research Campaign UK

**Effects of prostate inflammation on local and adjacent tissues**

**Ozgu Aydogdu, Patrik Aronsson, Michael Winder**

Department of Pharmacology, Institute of Neuroscience and Physiology, The Sahlgrenska Academy, University of Gothenburg, Gothenburg, Sweden.

**Introduction:** The concept of cross‐organ sensitization, where morbidity in one tissue leads to detrimental effects in adjacent tissues, is gaining recognition. Previous studies have demonstrated how prostate inflammation can lead to bladder overactivity and decreased compliance.^1,2^. However, the mechanism by which this occurs remains elusive. Currently, the aim was to study the effects of prostate inflammation on the innate contractile properties of the prostate, urinary bladder and urethra.

**Methods:** Male Sprague‐Dawley rats were administered an intraprostatic injection of either zymosan, leading to distinct local inflammation, or saline, serving as control. After 14 days, the animals were euthanised and the prostate, bladder and urethra were excised. Full‐thickness tissue strips were mounted in organ baths and the contractile responses to electrical field stimulation (EFS), methacholine, phenylephrine and ATP were examined.

**Results:** Induction of prostate inflammation did not alter innate prostate contractile responses to EFS, methacholine, phenylephrine or ATP. Likewise, no significant changes were seen regarding contractile responses in the isolated urethra. However, induction of prostate inflammation led to significantly decreased contractile responses to ATP in bladder strips.

**Conclusions:** The current findings show that induction of prostate inflammation can lead to changes in urinary bladder contractile properties. Surprisingly, no changes in the innate contractile properties of the prostate could be observed, strengthening the hypothesis that inflammation of the prostate can lead to LUTS not only because of local changes in the prostate but also because of induced changes in the bladder. The mechanism by which this cross‐organ sensitization occurs remains to be demonstrated.

**References:**

1. Aydogdu O, Uyar Gocun P, Aronsson P, Carlsson T, Winder M. Prostate‐to‐bladder cross‐sensitization in a model of zymosan‐induced chronic pelvic pain syndrome in rats. *Prostate* 2021; 81: 252–260.

2. Aydogdu O, Perez F, Aronsson P, Uyar Gocun P, Carlsson T, Sandner P et al. Treatment with the soluble guanylate cyclase activator BAY 60‐2770 normalizes bladder function in an in vivo rat model of chronic prostatitis. *Eur J Pharmacol* 2022; 175052.

**Ethical Approval:** University of Gothenburg, #4845/2024

**Funding:** The Colliander Foundation, the Wilhelm & Martina Lundgren foundation and the Royal Society of Arts and Sciences in Gothenburg.

**Urothelial contribution to nitric oxide relaxations in the healthy and inflamed rat bladder**

**Patrik Aronsson, Michael Winder**

Department of Pharmacology, Institute of Neuroscience and Physiology, The Sahlgrenska Academy, University of Gothenburg, Gothenburg, Sweden.

**Introduction:** We have previously demonstrated and validated our set‐up of a “nitric oxide (NO) in water” system, yielding a fixed concentration of NO in solution for several days.^1,2^ This set‐up was used to investigate nitrergic relaxatory pathways.

**Methods:** Presently, the functional role of reduced sGC in nitrergic relaxation is studied in controls and cyclophosphamide‐induced cystitis (100 mg/kg, 60 h before an experiment), using the sGC oxidizing agent ODQ (25 µM). The urothelial localization of sGC was assessed by denudation (collagenase 0.1% 30 min).

**Results:** Overall, ODQ reduced NO‐induced relaxation by 46‐58% in controls. This was statistically significant at the highest amount of NO (by 57.5%, i.e., from 48.9% to 20.8% of methacholine‐induced precontraction; 400 µL of NO solution; *p* = 0.0074). A similar reduction, but even more pronounced at lower NO concentrations, was observed in cystitis. Here, relaxation to all amounts of NO were significantly lowered by ODQ (by 82.7, 70.1, 49,6 & 39.9%; *p* < 0.05). Notably, the reduction in relaxation was greater at the lower concentrations. Removal of the urothelium seemed not to affect controls. Denudation did, however, reduce the NO‐induced relaxatory responses in inflamed tissues. Further administration of ODQ did not affect this (*p* > 0.05).

**Conclusions:** Oxidation of sGC with ODQ reduces NO relaxations, especially in inflamed bladder tissue. In the inflamed bladder, the target is likely largely located in the urothelium.

**References:**

1. Aronsson P, Stenqvist J, Ferizovic E, Danielsson E, Jensen A, Simonsen U et al. (2023). Soluble guanylate cyclase mediates the relaxation of healthy and inflamed bladder smooth muscle by aqueous nitric oxide. *Front Physiol*. 2023; 14: 1249560.

2. Simonsen U, Wadsworth RM, Buus NH, Mulvany MJ. (1999). In vitro simultaneous measurements of relaxation and nitric oxide concentration in rat superior mesenteric artery. *J Physiol*. 1999; 516, 271–282.

**Ethical Approval:** University of Gothenburg, #4845/2024

**Funding:** The Wilhelm & Martina Lundgren foundation and the Royal Society of Arts and Sciences in Gothenburg.

**Shear stress‐induced ATP release from isolated mouse urothelial cells**

**Basu Chakrabarty** ^**1**^, **Anthony J Kanai** ^**2**^, **Christopher H Fry** ^**1**^

^1^School of Physiology, Pharmacology & Neuroscience, Faculty of Health & Life Sciences, University of Bristol, Bristol, UK, ^2^Departments of Medicine and Pharmacology and Chemical Biology, University of Pittsburgh, Pittsburgh, USA.

**Introduction:** Sensory afferent nerves in the bladder are activated by transmitters like adenosine triphosphate (ATP) released from the urothelium when the bladder stretches during filling. Increased stretch‐activated ATP release has been linked to human bladder disease and animal models of bladder pathology.^1^ Previous studies demonstrate that increasing cyclic guanosine monophosphate (cGMP),^2^ or decreasing cyclic adenosine monophosphate (cAMP) through adenosine at A_1_ receptors^3^ can reduce neuronal ATP release in the mouse bladder. This study aimed to explore this paradigm in other bladder tissues, specifically the urothelium.

**Methods:** Urothelial cells were isolated from male C57BL/6 mice bladders and incubated at 36°C. Interventions (*n* = 6) were applied, and baseline ATP was measured. A series of 50 hydraulic shear loads, each 0.1 kPa, was imposed over 30 s. ATP release was measured 4 min poststimulus using a calibrated luciferin‐luciferase assay.

**Results:** The phosphodiesterase type‐5 inhibitor sildenafil (20 µM), which prolongs cGMP activity by reducing its breakdown, reduced shear force‐activated ATP release. The protein kinase G inhibitor, Rp‐8‐CPT‐cGMPS (10 µM), abolished the effect of sildenafil to reduce ATP release. Adenosine (1 mM) also reduced ATP release from urothelial cells, while the adenosine A_1_ receptor antagonist, DPCPX (1 µM), reversed the reduction of stretch‐activated ATP release by adenosine.

**Conclusions:** Increasing cGMP or decreasing cAMP levels reduces urothelial ATP release, which may potentially target purinergic sensory signaling pathways involved in bladder dysfunction.

**References:**

1. Grundy L, Wyndaele JJ, Hashitani H, Vahabi B, Wein A, Abrams P et al. How does the lower urinary tract contribute to bladder sensation? ICI‐RS 2023. *Neurourol Urodyn* 2023. doi:10.1002/nau.25316.

2. Chakrabarty B, Ito H, Ximenes M, Nishikawa N, Vahabi B, Kanai AJ et al. Influence of sildenafil on the purinergic components of nerve‐mediated and urothelial ATP release from the bladder of normal and spinal cord injured mice. *Br J Pharmacol* 2019; 176: 2227–2327.

3 Chakrabarty B, Drake MJ, Kanai AJ, Fry CH. Selective reduction of neurotransmitter release by cAMP‐dependent pathways in mouse detrusor. *Am J Physiol Regul Integr Comp Physiol* 2022; 323: R889‐R899.

**Ethical Approval:** University of Bristol, UIN 21/064

**Funding:** NIH R01 DK098361

## Author Contributions

Conceptualization, initial draft; CHF, final review and agreement to submit, all authors.

## Conflicts of Interest

The authors declare no conflicts of interest.

## Data Availability

Results represent preliminary outcomes, data are not yet available to third parties.

